# Seeding Structures for a Community of Practice Focused on Transient Ischemic Attack (TIA): Implementing Across Disciplines and Waves

**DOI:** 10.1007/s11606-020-06135-z

**Published:** 2020-09-01

**Authors:** Lauren S. Penney, Barbara J. Homoya, Teresa M. Damush, Nicholas A. Rattray, Edward J. Miech, Laura J. Myers, Sean Baird, Ariel Cheatham, Dawn M. Bravata

**Affiliations:** 1 Department of Veterans Affairs (VA) Health Services Research and Development (HSR&D), Precision Monitoring to Transform Care (PRISM) Quality Enhancement Research Initiative (QUERI), Indianapolis, IN USA; 2 VA HSR&D Elizabeth Dole Center of Excellence for Veteran and Caregiver Research, South Texas Veterans Health Care System, San Antonio, TX USA; 3 School of Medicine, University of Texas Health San Antonio, San Antonio, TX USA; 4 VA HSR&D Center for Health Information and Communication (CHIC), Richard L. Roudebush VA Medical Center, Indianapolis, IN USA; 5 Regenstrief Institute, Inc., Indianapolis, IN USA; 6 Department of Internal Medicine, Indiana University School of Medicine, Indianapolis, IN USA; 7 Department of Anthropology, Indiana University-Purdue University, Indianapolis, IN USA; 8 Department of Neurology, Indiana University School of Medicine, Indianapolis, IN USA

**Keywords:** situated learning, learning healthcare system, cerebrovascular disease, community of practice, implementation science, Veterans Health Administration

## Abstract

**Background:**

The Community of Practice (CoP) model represents one approach to address knowledge management to support effective implementation of best practices.

**Objective:**

We sought to identify CoP developmental strategies within the context of a national quality improvement project focused on improving the quality for patients receiving acute transient ischemic attack (TIA) care.

**Design:**

Stepped wedge trial.

**Participants:**

Multidisciplinary staff at six Veterans Affairs medical facilities.

**Interventions:**

To encourage site implementation of a multi-component quality improvement intervention, the trial included strategies to improve the development of a CoP: site kickoff meetings, CoP conference calls, and an interactive website (the “Hub”).

**Approach:**

Mixed-methods evaluation included data collected through a CoP attendance log; semi-structured interviews with site participants at 6 months (*n* = 32) and 12 months (*n* = 30), and CoP call facilitators (*n* = 2); and 22 CoP call debriefings.

**Key Results:**

The critical seeding structures that supported the cultivation of the CoP were the kickoffs which fostered relationships (key to the *community* element of CoPs) and provided the evidence base relevant to TIA care (key to the *domain* element of CoPs). The Hub provided the forum for sharing quality improvement plans and other tools which were further highlighted during the CoP calls (key to the *practice* element of CoPs). CoP calls were curated to create a positive context around participants’ work by recognizing team successes. In addition to improving care at their local facilities, the community created a shared set of tools which built on their collective knowledge and could be shared within and outside the group.

**Conclusions:**

The PREVENT CoP advanced the mission of the learning healthcare system by successfully providing a forum for shared learning. The CoP was grown through seeding structures that included kickoffs, CoP calls, and the Hub. A CoP expands upon the learning collaborative implementation strategy as an effective implementation practice.

**Electronic supplementary material:**

The online version of this article (10.1007/s11606-020-06135-z) contains supplementary material, which is available to authorized users.

## BACKGROUND

A key challenge for any learning healthcare system is to ensure knowledge is appropriately captured, shared, and used in support of implementation of effective practices.^1, 2^ Knowledge management becomes even more complex when implementation spans organizational units and disciplines.^3, 4^ Communities of Practice (CoP) represent one approach to address knowledge management in a way that may support effective implementation.^5^ CoPs are “groups of people who share a concern, a set of problems, or a passion about a topic, and who deepen their knowledge and expertise in this area by interacting on an ongoing basis.”^6^ CoPs have three critical elements: community, practice, and domain (Table 1). Within the CoP literature, learning is conceptualized as a social practice; individuals are engaged and develop a sense of belonging and identity.^7^

**Table 1:** Critical Elements of Communities of Practice (CoP)

Element	Definition
Practice	Members share experiences, tools, and ways of responding to similar issues that inform the way they practice.
Community	Members of a community engage in shared discussion and activities, share information, learn from/with each other, assist one another, and build relationships.
Domain	The area of shared interest to which members are committed and have competence. This element helps provide identity and meaning.

In health care, CoPs function in several ways, including social interaction, knowledge creation, knowledge sharing, and identity building.^8^ Multiple, large CoPs engage diverse providers across and within organizations to improve health care delivery.^9–14^ These efforts build on the strengths of CoP learning to achieve specific goals, and interaction is often virtual. Empirical studies of CoPs have described how they get started, their membership, their activities, how they communicate, and factors which influence their success,^15^ as well as the type of knowledge work that they perform^16^ and important roles their leadership play.^17^ Organizations can cultivate CoPs by providing “seeding structures” such as culturally symbolic infrastructure, infrastructure instruments, and conceptual points of focus.^18^

The current project builds on the existing CoP literature by evaluating strategies and seeding structures that enhanced the formation of a CoP focused on improving quality of care for patients with transient ischemic attack (TIA) in the US Veterans Health Administration (VHA) system.

## METHODS

### Objective

We sought to identify CoP developmental strategies within the context of a national quality improvement project focused on improving quality of care for patients receiving acute TIA care. This CoP was organized around a clinical domain that spans organizational units and disciplines but requires coordination across services to provide timely care: where there are typically very few individuals with topic expertise at a given medical center and where there is no system-wide quality measurement or management system.

### Design

The Protocol-guided Rapid Evaluation of Veterans Experiencing New Transient Neurological Symptoms (PREVENT) was a stepped-wedge trial to improve the quality of TIA care.^19^ VA facilities with identified gaps in guideline-concordant TIA care were invited to participate. A total of six facilities participated, with two facilities per wave, where a 1-year active implementation period was followed by a sustainability period. PREVENT is a registered trial (NCT02769338) and received human subjects approval from the Indiana University Institutional Review Board and the Richard L. Roudebush Department of Veterans Affairs (VA) research and development committee.

### Participants

Staff at six VA facilities, located in different regions across VHA, volunteered to participate in the trial. Led by a local champion, each facility developed a multidisciplinary local project team that planned and implemented local TIA care improvement projects to address local TIA care quality gaps.

### Interventions

The PREVENT intervention included five components: a quality care reporting system, clinical programs, professional education, electronic health record tools, and quality improvement support, including a CoP (see Appendix 1). An external facilitator provided tailored support across these components. Throughout the active implementation period, participating sites received monthly reports and data on the quality of TIA care at their facility across a set of guideline-concordant processes of care. Local teams developed site-specific strategies and processes for improving TIA care using (or not) the resources provided through the PREVENT program components. To support TIA care improvement, PREVENT included multiple tools to encourage relationship building, sharing, and learning among members of the CoP. Essential to this effort were in-person team kickoff meetings, an interactive website (the “Hub”), and monthly CoP calls (Appendix 1).

### Approach

This study draws on data collected as part of the PREVENT mixed-methods evaluation: CoP attendance log, semi-structured interviews, and CoP debriefs. An attendance log was maintained for each of the 22 CoP calls and contributions made by individual participants during the calls were noted. Attendance was descriptively analyzed by site, study phase (active implementation versus sustainability), and attendee role (champion versus other). After each of the 22 CoP calls, implementation team members met to debrief about the call (e.g., reviewing technical aspects, noting interactions among participants; Appendix 1). These debriefings were recorded and transcribed.

Semi-structured interviews were conducted with key stakeholders, the study principal investigator (PI), and the external facilitator (EF). Stakeholders were purposively and snowball sampled from staff involved in TIA care delivery at the six sites. Interviews were conducted by trained members of the implementation team. With verbal consent, all interviews were audio-recorded and transcribed verbatim. Interviews were conducted in-person or by telephone at 6 months and 12 months after initiating active implementation. The interview guide included questions related to participants’ perspectives on elements of the PREVENT intervention (see Appendix 2). Each interview transcript was independently coded in NVivo 12^20^ by two trained members of the implementation evaluation team. Final coding involved gaining consensus among the two coders. The overall project codebook was based on our implementation framework and emergent elements specifically related to PREVENT (e.g., External Facilitation, the Hub). For this analysis, using a hybrid thematic analysis approach,^21^ select interview code reports (e.g., collaborative calls, virtual collaborative), PI and EF interview notes, and debrief transcripts were reviewed (LP) and deductively sorted according to critical elements of CoPs (practice, community, domain (Table 1)). Categorization and inductively derived themes within each category were refined, and *a priori* thematic saturation^22^ agreed to, through discussions with the implementation team.

## RESULTS

### Participants

A total of 42 unique stakeholders were interviewed at 6 months (*n* = 32) and 12 months (*n* = 30) after initiation of active implementation; an average of 5 (range 3–8) staff were interviewed per site for each time period. Participants represented multiple disciplines; across sites, the most frequently represented groups were neurologists, emergency medicine physicians, pharmacists, and nurses.

### The PREVENT CoP: Critical Seeding Structures

**Figure 1:**
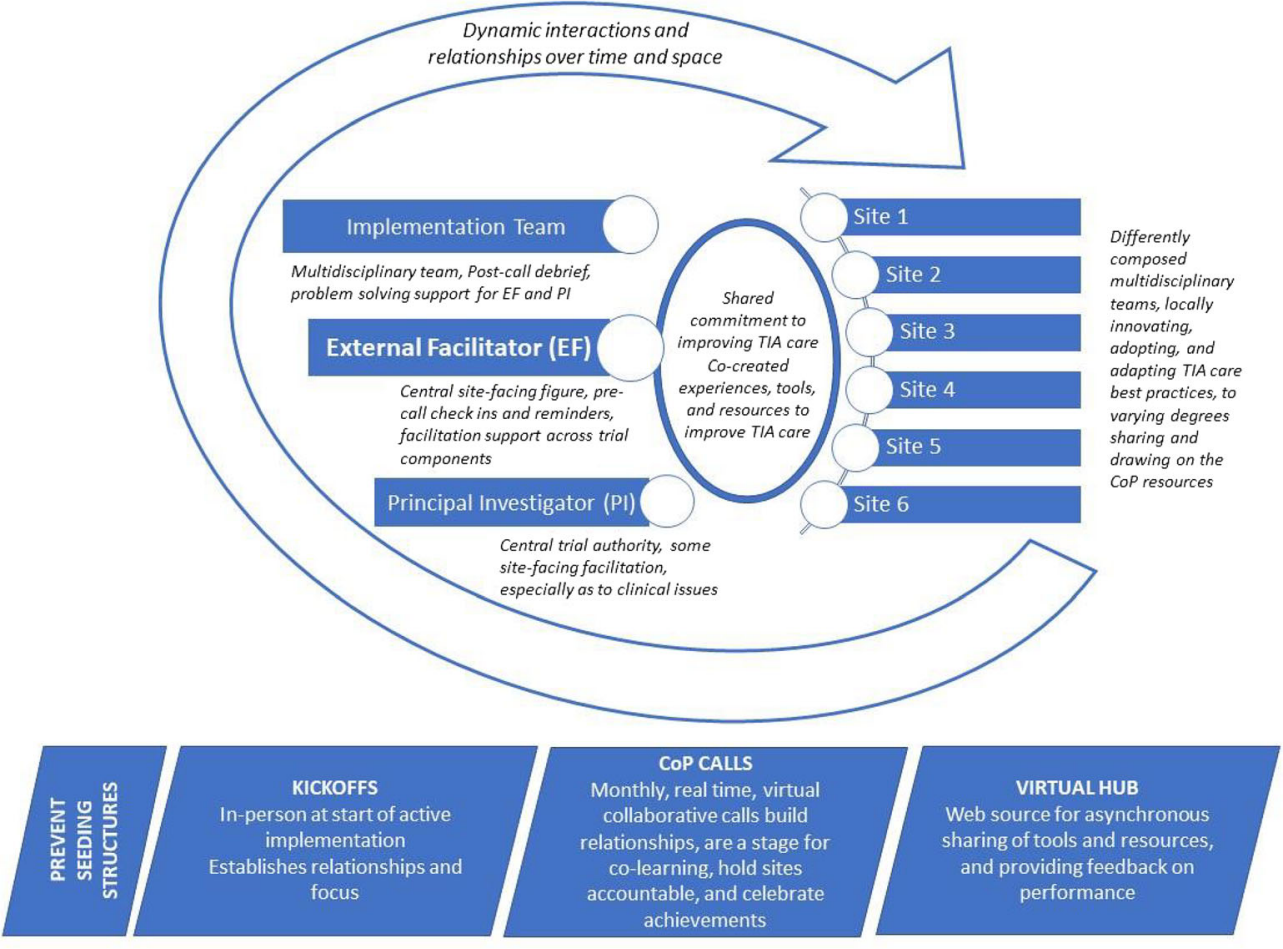
The PREVENT Community of Practice (CoP) and its critical seeding structures.

Development of the PREVENT CoP was a deliberative process built upon the focus and structure of the PREVENT quality improvement program. CoP calls were a curated and adaptable space for cross-organizational learning, sharing, and accountability. As shown in Figure 1, critical seeding structures that were foundational to the CoP were the kickoffs, the Hub, and the CoP calls; these were all explicitly designed as part of the PREVENT trial. The CoP included individuals from the participating sites and members of the implementation team. Relationships and plans established during the kickoffs gave meaning and purpose to the CoP (see Fig. 1). Relationships were enriched through use of the web-based Hub and, in particular, through the monthly CoP calls. On the Hub, group resources and tools could be shared. Performance metrics available on the Hub provided visual and social accountability for improvement. CoP calls also were a place to celebrate successes and inform identification with local and national organizations. Clinical champions were deliberately asked to present the achievements of their fellow peers in the CoP to further foster a community. Implementation team debriefs following CoP calls identified potential aspects for tailoring future sessions and opportunities for targeted external facilitation. The debriefs were essential for problem-solving challenges related to encouraging relationship-building between disciplines (within and across sites) and across a stepped wedge research design (where participants from sites in earlier waves have more time to develop relationships than sites in later waves).

The implementation team (see Fig. 1, upper left corner) strategically used and adapted elements during the kickoffs, CoP calls, and the Hub as seeding structures for the CoP (see Appendix 3) in an iterative process based on learning from previous waves. Throughout the project, during built-in team reflection periods, the implementation team identified and problem-solved challenges to community building, and tailored seeding structures, especially CoP calls, to enhance CoP development (Appendix 3).

### Practice

The PREVENT Hub and CoP calls had components which supported quality improvement, provided accountability, and celebrated achievements in effecting practice change. The implementation team used information collected during CoP calls to help tailor external quality improvement support.

The Hub was a CoP repository for shared resources, such as electronic medical record consult templates. Sites uploaded and shared their locally developed tools and also tailored shared tools for their own use. For example, one facility’s emergency department (ED) protocol and one facility’s patient brochure were each adapted and adopted by two other sites.

Participation on the CoP calls provided insights into how TIA care was provided across diverse organizational contexts. Teams at different medical centers implemented PREVENT in different ways and shared their creative efforts to improve TIA care (see Fig. 1, center circle). These CoP exchanges were valuable to other participants and could facilitate local problem solving:

It’s good to hear like what works well and what doesn’t work well or what challenges other VAs have had…listening to like how they overcame those challenges can really help us out, too because maybe we can piggyback off them and steal a few ideas. (104_12m_4)

One site neurologist described taking information from the CoP call devoted to medications and using it in a grand rounds presentation. In another example, after one facility team during a CoP call described their protocol for ensuring follow-up for TIA patients who leave the ED against medical advice (which presents a challenge for timely care), an ED physician at another site decided to adopt the protocol: “I said this is great. We have to bring it here” (102_12m_2). The protocol was also adopted by a site in a later wave. Towards the end of their active implementation phase, two sites considered adopting a site’s novel prospective use of the patient identification tool, which was discussed during many CoP calls as an effective, proactive strategy to ensuring timely care.

The CoP calls were also a place where the group could take different positions. Pragmatic discussion of case studies allowed debate on clinical “gray areas.” For example, a neurologist from one site presented a case during which a pharmacist from another site questioned the use of bile acid sequestrant, which led to a conversation about clinical uncertainty in lipid management for older patients.

Some CoP calls focused on different disciplines involved in TIA care (e.g., pharmacy medication management, the roles of nursing, and emergency medicine) allowing members to learn about best practices and experiences outside their discipline. For example, during one CoP call, a vascular surgeon and a neurologist discussed clinical care of patients with symptomatic and asymptomatic carotid stenosis from their different clinical experiences. For some, conversations like this brought a valued interdisciplinary awareness to their practice:

I’ve been in a few where the topics are very interesting even though it doesn’t have to do anything with radiology but sometimes, it’s good for us to actually get that management perspective as well. (104_12m_1)

Observing how other teams were configured also helped expand some participants’ understanding of the possibilities of multidisciplinary care:

I do remember initially when we were on one of the national calls, they had gone around and introduced all of the different sites, and I had heard about the makeup of each of those teams…having a multidisciplinary team that’s not the typical clinical team, I think that it has been very helpful to get that other perspective and then to then also get a better understanding of how other services are involved with the post follow-up care. (105_6m_3)

Other subject matter experts (e.g., systems redesign, implementation scientists) shared knowledge around other issues related to practice change:

We had that one that was about following through on goals once we’ve sort of graduated from a program. And so, I felt that that was very meaningful for us at that time. I really think that gave us some actual specific ideas or goals to try to keep momentum going and not have a drop off thing. (105_12m_4)

However, not all information or experiences on the CoP calls were perceived to be relevant. As noted above, sites were often at different stages of implementation due to the stepped-wedge design of the trial, with later wave sites having potentially more learning from earlier sites than vice versa. Participants, particularly clinicians, noted that leveling the knowledge bases across disciplines often meant that for some disciplines, discussions were less immediately useful:

I think that it’s tough to have one call meet the needs of people from multiple backgrounds… For the neurologists, it’s sort of like oh okay. Yeah. It’s not new information… it’s not to say that I don’t think that it’s useful. …When you’re having whole teams, there are probably portions of each team that find it useful…I think that for the neurology members of the team, it’s probably not useful. (105_6m_6)

In addition, when sites were perceived to be describing mundane updates that were not “outstanding or different,” that portion of the calls was sometimes perceived as less productive. Participants expressed similar sentiments if site activities did not seem directly relevant to their local context:

All of the facilities are so different. So we’re all implementing in very different ways. So like what they’re doing, their processes, I can’t say 100% that they’ve been super useful to me and… the same challenges that we face here. (103_6m_2)

Others acknowledged that with competing priorities, there were some inherent tensions:

It’s a fine balance between being a collaborative environment and being efficient and getting consensus … I’m looking for efficiency… I love all the feel-good stuff and I love hearing about what somebody did and all this stuff, but you know, it really boils down to what can you do for me. (103_6m_4)

### Community

**Figure 2:**
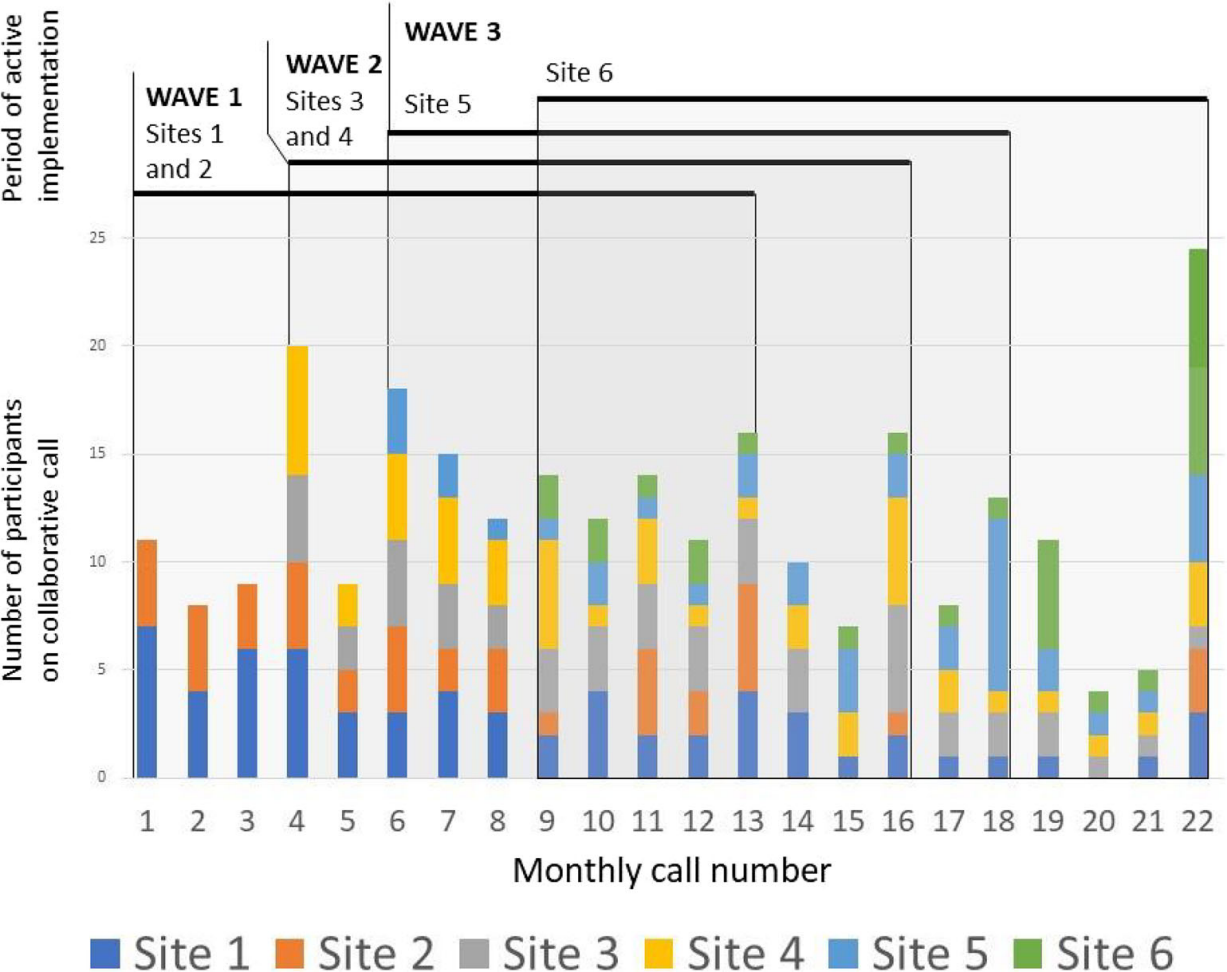
Monthly CoP call attendance by site.

The main tool for ongoing community building was the monthly CoP call. Participation on the monthly CoP calls varied over time, with an average of 12 people from the sites present during any one call. A total of 61 unique individuals participated in at least one CoP call (mean 10 staff per site, range 7–16; see Fig. 2). Each site had a core group of 1–3 participants who attended the majority of CoP calls during their site’s 12-month active implementation (teams typically included 3–6 individuals). The first and last calls during active implementation were especially well-attended. Attendance on CoP calls after the end of active implementation was less regular; 13 individuals attended at least one call after their site completed active implementation (average 2.23 calls, range 1–5 calls). Participants represented a diversity of disciplines, including neurology physicians (27% of total attendance), pharmacists (21%), nurses (17%), and medicine physicians (17%).

Participation peaked on the final call (24 participants), which featured the promotion (i.e., end of active implementation) of site 6 and a reunion for all sites. The smallest turnout was on the twentieth call (4 participants), when only site 6 was still in active implementation. The second most-attended call was the fourth call, when sites 3 and 4 entered active implementation.

A key challenge to community building was the stepped-wedge design of the PREVENT trial. During the first wave, only two sites were participating in PREVENT, which promoted the growth of relationships between team members from the two wave-1 sites (Fig. 2). As additional waves were added, the community shifted from an intimate group to a larger (“national”) group; the degree to which people expressed a sense of affiliation with the group varied across individuals and time:

I guess (I felt) more (of a sense of community) in the beginning when it was just a few sites. More recently not quite so much. … maybe it wasn’t that it was just a few sites. It was just that we were very active at that time. So I felt more of a community. (102_12m_3)

Membership shifted with each new wave of implementation. Providing equivalent time for growing numbers of the PREVENT community also created pressures on the call, as providing individualized attention took away from time otherwise devoted to educational matters and group discussion. As new sites came on, some participants noted they were getting less out of the call:

It’s a multisite project, and you’ve got to do a lot of or a little bit of cheerleading for your people, and you’ve got to do that for every site, and as more and more sites come on board, you’re spending more time cheerleading than I'm getting information back for myself to improve our process here. (103_12m_4)

Call participation was generally small enough so that people could potentially recognize one another’s names and voices, and have small group discussion. People contributed to the calls, both verbally and through the chat box, in different ways: providing site updates, asking questions or providing suggestions related to implementation, weighing in on clinical cases or providing content expertise, and providing a listening audience. Some individuals had more apparent comfort with speaking up during calls. Across calls, team champions (e.g., nurse and neurologists) spoke up most often. Other team members were more likely to talk (and specifically prompted to talk) when call topics touched on their area of practice. Not all efforts to inspire engagement were effective. For example, facilitators attempted to elicit “burning questions” and ideas for educational topics from sites but sometimes received few suggestions.

Discussions about sites’ experiences created a sense of common enterprise (see Fig. 1, center circle), as well as providing perspective and insights about their own sites, that was widely valued by participants:

It’s nice to hear that other people have similar questions to what you have when you’re on the calls, so I think that’s, you should definitely keep those up, because I think it kind of creates a bigger cohesive group. (103_6m_6)

Hearing the experience at other places…Kind of hearing their starting point and then their challenges. Some of it I could be like yeah, we had the same problems. Some of them I'm like woo. I’m glad I don’t have that problem (laughter)…To some extent, we’re all in the same boat. But sometimes our boat might be slightly better than somebody else’s boat. (104_6m_3)

Participants generally recognized that the external facilitator (see Fig. 1) encouraged participation and that the calls were safe spaces to genuinely share their own perspective:

I think that they get us all involve[d]. Like even at the last phone call, they were going over the case, and they were asking people’s opinion. … I feel like this is one of the meetings that if I feel like I have something to say, like when I get on the line, I'm not going to get crucified for it. (102_6m_3)

### Domain

PREVENT participants described how participating in the CoP brought meaning to their work. They expressed a common sense of mission and implied that this effort both reinforced and created new facets to their professional identifies. However, promoting group coherence and identification across time, given differing priorities and experience, was a challenge.

Team kickoff meetings established the foundation for the CoP. Kickoffs provided a sense of purpose and helped build relationships and trust within the local team and between the project participants and implementation team which were then expanded upon through the monthly CoP calls (see Fig. 1).

[The kickoff] got everybody on board…it gave us that time to be able to focus on what the problem was…kind of got everybody on the same page…the fact that you guys came on site emphasizes or heightens the importance of it. (101_6m_6).

CoP calls sometimes featured national level leadership which created a sense of a larger, institutional mission. Across sites, participants expressed a feeling of being part of a larger, specialized community to improve TIA care, and being at the forefront of the field:

I do call in and listen, touch base, and see how things are going and listen to the educational sessions… I think that they’re very good for those that are highly vested in stroke PREVENT TIA… Because there is a little bit of a passion there anyway… you get good information out of them and it stays on top of the trends and where we’re going. (101_12m_1)

The CoP calls also reinforced aspects of participants’ professional identities, such as stroke care experts, care improvers, organizational members, and local site representatives. The implementation team was attentive to topics in which people were passionate and prompted them to participate during calls.

## DISCUSSION

Over the course of the PREVENT program, a CoP formed around the goal of improving TIA care, incorporating the three critical elements of CoPs: *practice*, *community*, and *domain*. The CoP was created through careful, yet flexible organization, agenda-setting, and facilitation. The critical seeding structures that supported the CoP were the kickoffs which fostered relationships (key to the *community* element of CoPs) and provided the evidence base relevant to TIA care (key to the *domain* element of CoPs). The Hub provided the forum for sharing quality improvement plans and other tools which were further highlighted during the CoP calls (key to the *practice* element of CoPs). CoP calls were curated to create a positive context around participants’ work by recognizing team successes. In addition to improving care at their local facilities, the community created a shared set of tools (e.g., templates, reports, dashboards) which built on their collective knowledge and could be shared within and outside the group. While interrelated with all PREVENT program components, Figure 1 illustrates how the CoP was a medium through which participants interacted with the PREVENT program, and uniquely facilitated participants’ sense of meaning and connection, engagement in collaborative learning, and feeling of social accountability. The PREVENT CoP provided a real-life example of what a “learning healthcare system” looks like in practice.

PREVENT program characteristics that encouraged CoP development, including an adept external facilitator, a focus on specific TIA care processes and metrics, in-person project kickoffs, committed and engaged voluntary local champions, support from relevant organization leaders, organized and routine group interaction, and a shared set of knowledge and tools, overlap with previously identified determinants of success for CoPs.^15^ In addition to these factors, PREVENT offered participants an opportunity to create solutions together and have their expertise and efforts recognized. The CoP had multiple feedback loops that allowed the implementation team to tailor content and organization of the PREVENT program to encourage uptake of best practices, with built in social accountability for participation and progress. These positive characteristics may be useful guideposts for future quality improvement programs using CoP as a time delimited strategy for encouraging the uptake of guideline concordant care.

Although PREVENT offered tools and data, sites were encouraged to create locally tailored implementations. CoP call topics typically touched on cutting edge of current TIA-related care and/or health system improvement practices. Debates about clinical guidelines engaged participants and provided space for co-exploration and thinking together.^23^ Those spontaneous discussions were perceived to be key to encouraging ongoing attendance. In a crowded marketplace of information about best practices for clinical care which might otherwise discourage engagement in a CoP,^24^ using an integrated set of strategies,^25^ the PREVENT CoP offered an interactive, unique space for learning. As opposed to trying to determine relationships and specific practices *a priori*, PREVENT provided what Thompson (2010) has referred to as seeding infrastructure: a conceptual point of focus (TIA care) and elements of infrastructure (CoP calls, the Hub) around which people could organize and allow their local program to flourish. Moreover, the PREVENT CoP expanded upon the Expert Recommendations for Implementing Change [ERIC] implementation strategy “learning collaborative” with all of its seeding infrastructure.^26^

Debriefs after CoP calls with the PREVENT implementation team allowed for ongoing refinement to improve future calls. Regular feedback from participants was also key to ensuring that the CoP was meeting participants’ needs and agendas^27^

Most participants returned to the CoP calls each month because they were an opportunity to learn. The external facilitator and PI served as knowledge brokers who brought together often siloed groups^28, 29^; they shaped the narrative around appropriate and high quality TIA care, while also opening that conversation up to group debate, especially around gray areas of best practices. Although the evidence for best practices could be bound up in different specialties, PREVENT forged a new CoP that spanned multiple disciplines and sites by constructing opportunities for building relationships and sharing knowledge (see also^27^). This experience is relevant to healthcare systems, like the VA, with distributed expertise across sites and specialties. CoPs with structures like PREVENT may offer organizations new ways to effectively bridge interdisciplinary knowledge and practice gaps to effect interdisciplinary care and improve patient outcomes.

The CoP calls also served to provide accountability and reinforce positive practice change. Having a public progress report every month motivated teams to meet before calls to coordinate and plan. Promotion ceremonies, which involved invited local and national level leadership, strategically used personalization and praise to foster a sense of professional satisfaction, connection to the organization, motivation to participate, and reinforce improvement efforts. By participating in a national CoP and working to improve care for patients beyond their local medical center, members of a CoP reported feeling being part of something greater than themselves.

Several limitations of this study should be considered. First, because the CoP was part of a complex, integrated QI program, we cannot isolate its specific effects independent from other program components. Second, participants in the PREVENT CoP were volunteers who agreed to work on a specific quality problem; the results may not be generalizable to CoPs with more diverse focus areas or that are centrally mandated. Third, all participants were VA staff working within VA facilities and under the same national leadership; results may not generalize to non-VA settings where participants may not have baseline affiliation, common points of reference and tools, and similar organization of care and authority figures. Fourth, the CoP members were multidisciplinary; therefore, some of the observed challenges may not be relevant to CoPs within a specific professional discipline. Fifth, the PREVENT CoP was developed in the setting of a trial with a principal investigator; it is not clear how well these findings would translate to a quality improvement project helmed by an organizational leader or manager. Finally, participants were expected to join the CoP for the 1-year active implementation phase of the PREVENT study and hence, it was expected that attendance decreased during the sustainability period. Therefore, the observed attendance pattern is unlikely to be representative of CoPs that are not embedded within a stepped-wedge trial design. Future research should evaluate how the state of knowledge within the field impacts CoP development and how interdisciplinary power dynamics influences participation.

In conclusion, the PREVENT CoP advanced the mission of the learning healthcare system by successfully providing a forum for shared learning. As visualized in Figure 1, the CoP expands upon the learning collaborative implementation strategy by building on a foundation of seeding structures that included kickoffs, CoP calls, and the Hub. Healthcare organizations that seek to support CoPs should consider including routine reflection and evaluation of the CoP to ensure that it enables members to cultivate their own quality improvement programs.

## Electronic supplementary material

ESM 1 (DOCX 22 kb)
