# Supplementary material for: Seeding Structures for a Community of Practice Focused on Transient Ischemic Attack (TIA): Implementing Across Disciplines and Waves
Source: J Gen Intern Med. 2020 Sep 1;36(2):313–21. doi: 10.1007/s11606-020-06135-z (PMC7878647; doi:10.1007/s11606-020-06135-z)
Supplement: Supplementary file 1 — (DOCX 22 kb) [file 11606_2020_6135_MOESM1_ESM.docx]

## Appendices

**Appendix 1. PREVENT Program and Tools Related to the CoP Formation**

The Protocol-guided Rapid Evaluation of Veterans Experiencing New Transient Neurological Symptoms (PREVENT) trial aimed to encourage participating sites to develop local QI projects that would result in the timely delivery of guideline concordant transient ischemic attack (TIA) care. Sites were given flexibility in terms of tools, processes, and/or policies they implemented to address quality of care gaps.

Quality of care was assessed based on site overall “without fail rate” and the timeliness of delivery of seven guideline concordant processes of care. The without fail rate assessed the proportion of Veterans with TIA who received all of the processes of care for which they were eligible. The seven processes of care included brain imaging, carotid artery imaging, hypertension medication intensification, neurology consultation, hypertension control, anticoagulation for atrial fibrillation, antithrombotics, and high- or moderate-potency statin therapy. These processes of care were often the target of local site QI work. The PREVENT program included five components aimed to help sites overcome barriers to implementation: a quality of care reporting system (e.g., monthly performance data, quarterly outcome data), clinical programs (e.g., post-discharge nurse follow-up tool, Emergency Department protocols), professional education (e.g., pocket cards, targeted neurology education), electronic health record tools (e.g., patient identification tool, order menus), and QI support (e.g., strategic planning support, monthly community of practice virtual call). These served as resources for sites as they problem solved their local quality of care gaps. The PREVENT program components were delivered through multiple mediums, including three specific tools that were used to build relationships and facilitate learning between sites: in-person team kickoff meetings, an interactive website (the “Hub”), and monthly CoP calls (see table below). These were interrelated elements within the PREVENT program.

| **PREVENT Tool** | **Description** | **Related PREVENT Program Component(s)** |
| --- | --- | --- |
| Kickoff | Occurred at each site, one month before active implementation  In-person team kickoff meetings included local multidisciplinary team, external facilitator (BH), Principal Investigator (DB), and 6-8 members of the implementation team  All day meetings included team building activities, PREVENT program overview, site data review, identification of key opportunities for improvement, problem-solving, and action plan development. Members of the implementation team facilitated different portions of the program, depending on their expertise | Quality of care reporting system  Quality improvement support |
| The Hub | Web-based resource for TIA care quality improvement. Data updated each month for all facilities within the VA.  The external facilitator provided office hours one-on-one assistance to support use of data and other resources on the Hub.  Contains site-specific data related to care processes, healthcare utilization, and other aspects of care, along with VA averages and recommended targets. Other resources include articles, QI tools, and archived CoP call products (e.g. PowerPoints). Teams were required to upload and share their project action plans, and encouraged to share other tools (e.g. patient education documents). | Quality of care reporting system  Clinical protocols  Professional education  Electronic health record tools  Quality improvement support |
| CoP Call and Debriefment | Occurred monthly during the three waves of active implementation (for a total of 22 times from August 2017-May 2019)  Virtual, Skype for Business meeting for all participants whose sites had started active implementation. Special site promotion calls at the end of site active implementation and a final reunion call were hosted on a VA video teleconferencing system.  Calls were hosted and organized by the external facilitator (BH) and study Principal Investigator (DB).  Hour-long call formats varied, but generally included introductions, updates from sites in active implementation, and a learning component and discussion. Each call had a topical focus (see table below), aimed at improving knowledge of best practices in TIA care (e.g. statin guidelines) and/or local capacity for quality improvement (e.g. reflecting and evaluating). The calls qualified for continuous medical and nursing education credit.  Post-call debriefs by the implementation team covered logistical issues that could be improved (e.g., technology malfunctions, timing), evaluated site progress (e.g., goals met, challenges), and identified places for targeted external facilitation (e.g., updating resource materials to better reflect current guidelines, sharing tools with site champions). | Professional education  Quality improvement support |

**Appendix 2. Sample of Relevant Stakeholder Interview Questions**

DURING THE PAST 6 MONTHS: How often have you attended the monthly calls? What information from those calls has been the most helpful to you? Least helpful? Why: could you give an example?

What are your thoughts about hearing updates from other facilities? Have you been able to adapt any of the other teams’ protocols or materials to your local PREVENT program? If so, which and how did it turn out? Do you have any future plans to implement projects or protocols that have been developed by other VAMCs participating in PREVENT?

· Have you had direct communications with another PREVENT team member from another VA facility? Who? How often? What motivated you to reach out?

· Have other PREVENT teams reached out to you? If so, what was the reason?

· Are you involved in any cerebrovascular professional organization? Any other VA collaborative – Emergency Medicine, Pharmacy, Nursing? If so, how often does the group meeting and by what mode (Virtual, Telephone, In person meeting)?

· How much do you feel part of a community of practice for TIA care? [prompt: Do they think they have gotten to know the PREVENT participants/staff? More so than a general listserv?

· Did the calls increase your utilization of the hub?

· Did the calls create a sense of profession community?

· Did the calls serve to maintain your interest and enthusiasm about TIA quality improvement?

Were you able to use any of the existing PREVENT materials provided by the national program? Which ones? How did you adapt to your local facility? How helpful was it for your team to have access to the shared PREVENT materials? How often did you share your PREVENT materials and program with your peers at your facility? What has been the reaction of your peers to the PREVENT program at your facility?

**Appendix 3. Tailored Seeding Structures to Enhance CoP Development and Function**

| **Challenge** | **Solution** | **Description** |
| --- | --- | --- |
| Leveraging the strengths and expertise of the implementation team | Distribute roles, create spaces for team reflection | EF was the provider-facing navigator across PREVENT components (especially for the CoP calls, which she hosted, and Hub)  PI had a less defined role on calls, frequently provided broader context and clinical relevance for what was discussed and asked questions to specific members of the group and guest presenters to elicit expertise  Other members of the PREVENT implementation team played more or less visible critical roles, including the project coordinator who managed the logistics of calls and the data scientist who frequently guested on calls to provide information about PREVENT metrics  Engaged all members of the implementation team in debriefs after team kickoffs and CoP calls to identify challenges and successes, and problem solve |
| Providing effective resources for change | Identify areas with greatest potential for impact across content areas | The initial list of call topics was based on care processes believed to have the biggest opportunities for improvement (e.g. statins). Other topics provided more general information important to facilitating process improvement (e.g., a primer in implementation science, how to make a business case for more resources to leadership). |
| Gaining participant confidence for evidence-basis of recommended care processes | Provide evidence, draw on experts, allow for critical reflection | Calls often directly addressed the evidence for TIA and other related practices, included experts from the participating sites, the broader VA, and VA leadership. Time was also allotted for group reflection of the evidence and for coming to consensus around it. |
| Ensuring call productivity | Tight choreographing and preparation work | EF and PI set specific objectives for each call. Call preparation work (including gathering materials from presenters and site champions, preparing responses to site updates, doing run throughs to test equipment and timing), assured efficiency in meeting goals for each call. |
| Encouraging CoP call attendance | Multiple rounds of outreach, personalization | EF encouraged participation through multiple rounds of pre-call personalized invitations and reminders, and built trust, connections, and familiarity using chit chat at the start of calls and active listening throughout calls |
| Facilitating co-learning across disciplines | Identify and elicit group multidisciplinary expertise | Call facilitators identified participants who could speak to call topics and invited their contributions, and during debriefs discussed who had been engaged and who had not. Efforts were made to highlight the contributions of all members of the interdisciplinary teams. |
| Facilitating co-learning across multiple trial waves | For later waves, create different takes on early call content, create alternative ways to disseminate information (office hours, archives) | Separate facilitation office hours were created to help champions in later waves navigate the Hub.  Early call topics (e.g. hypertension) were repeated during later waves, but with different presenters, with slightly different takes. All calls were archived on the Hub to increase accessibility. |
| Nurturing personal investment in and identification with PREVENT | Personalization | Referring to people by name and incorporating levity into calls  Showing team pictures during site updates to promote relationships |
| Focusing team energies around project goals | Social accountability for goals | Calls had multiple accountability components that emphasized attention to goals. Prior to each call, the EF elicited site progress updates and formatted them into PowerPoint slides that site representatives used as call talking points (e.g. an area of progress, current goals). EF further reinforced goals by emailing each team immediately after calls and about two weeks before the next call. |
| Identifying gaps in understanding or implementation challenges | Monitor calls to identify problems and group problem-solve interventions | The implementation team observed calls to identify implementation challenges and strategized approaches to help a site(s) overcome those challenges (e.g., personal outreach from the EF, linking sites to resources, creating a CoP call to address the issue). |
| Encouraging positive change | Celebrate achievements, peer and leadership recognition | The EF periodically used the Hub news section to highlight site successes.  End of active implementation promotion ceremonies were an additional source of personalization, positive reinforcement, and motivation, and intended to encourage collegiality and professional pride. Call facilitators reviewed site successes and activities. Team members could share what participation meant to them both personally and professionally, and were handed diplomas (signed by the VA’s Chiefs of Emergency Medicine and Neurology Service) from their site leadership and were shipped homemade cookies by the implementation team. The EF enabled a peer from another participating site to identify and describe those achievements (“accolades”) during the celebration. |
